# Analytical Solutions of a Two-Compartment Model Based on the Volume-Average Theory for Blood Toxin Concentration during and after Dialysis

**DOI:** 10.3390/membranes11070506

**Published:** 2021-07-05

**Authors:** Yoshihiko Sano, Kentaro Sato, Ryusei Iida, Narutoshi Kabashima, Toyomu Ugawa

**Affiliations:** 1 Department of Mechanical Engineering, Shizuoka University, 3-5-1 Johoku, Naka-ku, Hamamatsu 432-8561, Japan; sato.kentaro.16@shizuoka.ac.jp (K.S.); fizzwater92@gmail.com (R.I.); 2 Hibiki Clinic 3-2-2, Korosuekita, Mizumaki-Machi Onga-Gun, Fukuoka 807-0022, Japan; nkaba@mocha.ocn.ne.jp; 3 Department of Advanced Systems Modeling in Intensive Care Medicine, Tokyo Medical and Dental University, 1-5-45 Yushima, Bunkyo-ku, Tokyo 113-8510, Japan; ugawat@gmail.com

**Keywords:** analytical solution, dialysis treatment, two-compartment model, volume-average theory

## Abstract

Accurate prediction of blood toxin concentration during and after dialysis will greatly contribute to the determination of dialysis treatment conditions. Conventional models, namely single-compartment model and two-compartment model, have advantages and disadvantages in terms of accuracy and practical application. In this study, we attempted to derive the mathematical model that predicts blood toxin concentrations during and after dialysis, which has both accuracy and practicality. To propose the accurate model, a new two-compartment model was mathematically derived by adapting volume-averaging theory to the mass transfer around peripheral tissues. Subsequently, to propose a practical model for predicting the blood toxin concentration during dialysis, an analytical solution expressed as algebraic expression was derived by adopting variable transformation. Furthermore, the other analytical solution that predicts rebound phenomena after dialysis was also derived through similar steps. The comparisons with the clinical data revealed that the proposed analytical solutions can reproduce the behavior of the measured blood urea concentration during and after dialysis. The analytical solutions proposed as algebraic expressions will allow a doctor to estimate the blood toxin concentration of a patient during and after dialysis. The proposed analytical solutions may be useful to consider the treatment conditions for dialysis, including the rebound phenomenon.

## 1. Introduction

**Figure 1:**
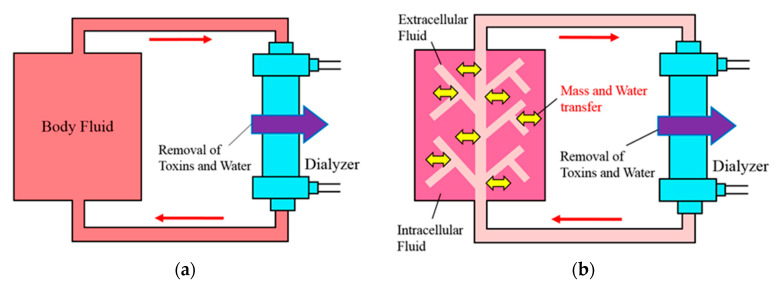
Pool model images for assessing the toxin concentration in the body during dialysis; (**a**) shows single-compartment model for which a whole body is a contaminated pool, while (**b**) shows the two-compartment model where the body is divided into intracellular and extracellular fluids.

The standardized *Kt*/*V*, which indicates the ratio of blood purification amount by dialysis to the amount of body fluid for the patient, is widely used today as an index to evaluate dialysis adequacy [1,2]. The standardized *Kt*/*V* consists of three important dialysis parameters, namely, *K*: clearance (mL/min), *t*: dialysis time (min), and *V*: total volume of body fluid (m^3^) for the patient. This standardized *Kt*/*V* was proposed by Gotch and Sargent [1] for predicting the blood toxin concentration during dialysis. They assumed that a whole body is a contaminated pool (single compartment) and a loop for blood purification is connected during dialysis treatment (see Figure 1a). Their model was simple and acceptable to doctors who treated with dialysis at the time, however, the model did not consider the effect of the volume decrease by ultrafiltration and toxin production in the body. Subsequently, several improved models have been proposed, so that the Daugirdas formula is recommended by the K/DOQI guidelines; on the other hand, the Shinzato formula is recommended by the Japanese Society for Dialysis Therapy [3,4,5]. However, their models are also proposed based on single-compartment assumption.

Pedrini et al. [6] raised the question of the validity of the models derived by the single-compartment assumption. Subsequently, some models taking into account the non-uniformity of metabolic substances in the body have been proposed [7,8]. There is the two-compartment model [8] in them, which was proposed based on an assumption that the body consists of intracellular and extracellular fluids (see Figure 1b). In this model, a loop for blood purification is connected at an extracellular fluid, and mass transfer takes place between intracellular and extracellular fluids. This model was a more realistic model because blood purification in dialysis is performed via blood. On the other hand, this model was more complicated than the single-compartment model. Some expression formulas [8,9,10,11,12,13,14] have been proposed as two-compartment models so far, and none were mathematically modeled from mass transfer in the body. Furthermore, in almost these studies, numerical simulation was used to evaluate the blood toxin concentration. Similarly, there is peritoneal dialysis as a treatment method for purifying blood. In peritoneal dialysis, the mass transfer takes place between multiple compartments such as blood, peritoneum, and dialysate. Several multi-compartment models have been proposed so far [15,16,17,18], which have been evaluated both numerically and analytically. Generically, numerical simulation requires considerable calculation time and is less accurate than an analytical solution. In contrast, an analytical solution is an algebraic expression, so that we can obtain the blood toxin concentration in any time period and any conditions when substituting dialysis conditions. Furthermore, the analytical solution can be implemented into the dialysis device, and the doctor may perform hemodialysis by predicting the blood toxin concentration.

In the present study, we attempted to derive the mathematical model that predicts blood toxin concentrations during and after dialysis, which has both accuracy and practicality. First, the new two-compartment model was mathematically derived by adapting volume-averaging theory to the mass transfer around peripheral tissues. Volume-averaging theory is a kind of multi-scale theory that derives a macroscopic governing equation from a microscopic governing equation by coupling heat or mass transport phenomena in numerous small elements existing in a large space [19,20,21,22,23]. Subsequently, analytical solutions for a two-compartment model were derived as a practical model for predicting blood toxin concentrations during and after dialysis. The post dialysis analytical solution for predicting the post dialysis rebound phenomenon was derived from the same procedure. The validity of the proposed solutions was examined by comparing with clinical data and numerical solutions. Furthermore, the proposed analytical solutions were compared with Gotch formula [1] and Shinzato formula [4], which are the single-compartment models.

## 2. Theory and Analytical Solutions

**Figure 2:**
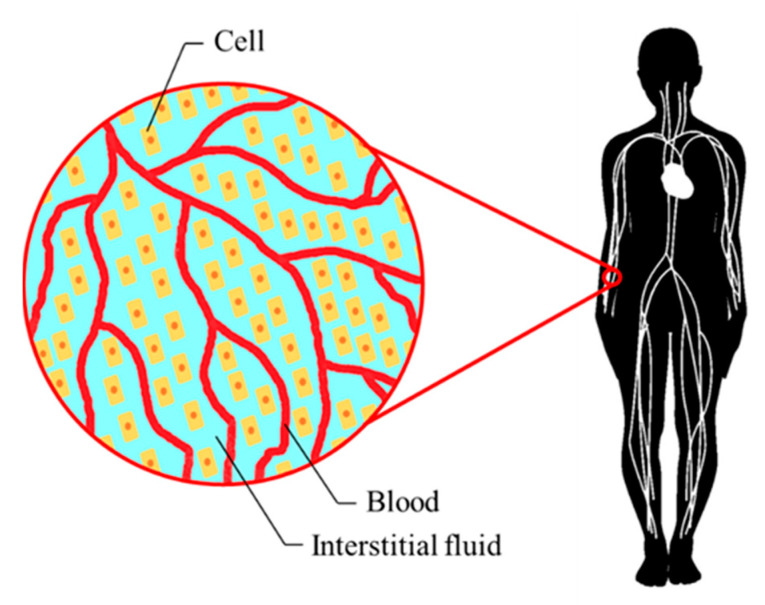
Analysis object viewing peripheral tissues as porous media. In the present study, mass transfer between extracellular and intracellular fluid phases is considered.

We shall consider the mass transport phenomena around peripheral tissues, as shown in Figure 2. According to Pedrini et al. [6], the toxin concentration differs mainly inside and outside the cell, because mass transfer resistance at the cell membrane is higher than that of the capillary wall. Therefore, in the present study, we focus on the toxin concentrations in extracellular and intracellular fluid phases and consider the mass transfer between theses phases. Note that active transport via ion channels is not considered in the present study.

In volume-averaging theory, the intrinsic volume average of a certain variable *ϕ* is defined as:

(1) $${\langle \phi \rangle }^{phase}=\frac{1}{V_{phase}}\int_{V_{phase}}\phi dV$$

where *V_phase_* is the volume occupied by intracellular and extracellular fluids. When applying volume averaging theory, considering the control volume scale [22], the control volume scale *V* in Figure 2 should be much greater than the microscopic characteristic length (cell length), but at the same time, should be much smaller than the macroscopic characteristic length (human length). Furthermore, a variable is decomposed into its intrinsic average and the spatial deviation therefrom:

(2) $$\phi ={\langle \phi \rangle }^{phase}+\tilde{\phi }$$

In volume-averaging theory, the following spatial average relationships are used (for details, see [19,20,21,22,23]):

(3a) $${\langle \phi_{1}\phi_{2}\rangle }^{phase}={\langle \phi_{1}\rangle }^{phase}{\langle \phi_{2}\rangle }^{phase}+{\langle {\tilde{\phi }}_{1}{\tilde{\phi }}_{2}\rangle }^{phase}$$

(3b) $${\langle \frac{\partial \phi }{\partial x_{i}}\rangle }^{phase}=\frac{1}{\varepsilon_{phase}}\frac{\partial \varepsilon_{phase}{\langle \phi \rangle }^{phase}}{\partial x_{i}}+\frac{1}{V_{phase}}\int_{A_{\mathrm{int}}}\phi n_{i}dA$$

(3c) $${\langle \frac{\partial \phi }{\partial t}\rangle }^{phase}=\frac{\partial {\langle \phi \rangle }^{phase}}{\partial t}$$

where *ε_phase_ = V_phase_/V* is the local volume fraction of each space, and *A_int_* is the interfacial area between each phase within the control volume. In the present study, *n_i_* is defined as a unit vector pointing outward from the extracellular fluid to the intracellular fluid.

The microscopic species mass transfer equations for the individual phases can be written as follows:

(4) $$\frac{\partial c}{\partial t}+\frac{\partial u_{j}c}{\partial x_{j}}=\frac{\partial }{\partial x_{j}}\left(D_{phase}\frac{\partial c}{\partial x_{j}}\right)+s$$

where *D* is the diffusion coefficient, and the species concentration *c* can be treated as an independent component in metabolic substances, such as urea nitrogen or creatinine. Furthermore, *s* indicates the toxin production rate per unit volume, which is assumed as a constant value and it would be zero extracellular fluid phases. The *x_j_* indicates Cartesian coordinate, which has *x*, *y* and *z*. The index *j* is a subscript of Einstein notation, which represents from 1 to 3.

The macroscopic governing equations for the extracellular and intracellular phases can be expressed using the above spatial average relationships:

(5) $$\begin{array}{l} \frac{\partial \varepsilon_{ex}{\langle c\rangle }^{ex}}{\partial t}+\frac{\partial \varepsilon_{ex}{\langle u_{j}\rangle }^{ex}{\langle c\rangle }^{ex}}{\partial x_{j}}\\ =\frac{\partial }{\partial x_{j}}\left(\varepsilon_{ex}D\frac{\partial {\langle c\rangle }^{ex}}{\partial x_{j}}+\frac{D}{V}\int_{A_{{}_{\mathrm{int}}}}cn_{j}dA-\rho \varepsilon_{ex}{\langle \tilde{c}{\tilde{u}}_{j}\rangle }^{ex}\right)+\frac{1}{V}\int_{A_{{}_{\mathrm{int}}}}D\frac{\partial c}{\partial x_{j}}n_{j}dA+\frac{1}{V}\int_{A_{{}_{\mathrm{int}}}}cu_{j}n_{j}dA \end{array}$$

(6) $$\frac{\partial \varepsilon_{in}{\langle c\rangle }^{in}}{\partial t}=\frac{\partial }{\partial x_{j}}\left(\varepsilon_{in}D\frac{\partial {\langle c\rangle }^{in}}{\partial x_{j}}-\frac{D}{V}\int_{A_{{}_{\mathrm{int}}}}cn_{j}dA-\rho \varepsilon_{in}{\langle \tilde{c}{\tilde{u}}_{j}\rangle }^{in}\right)-\frac{1}{V}\int_{A_{{}_{\mathrm{int}}}}D\frac{\partial c}{\partial x_{j}}n_{j}dA-\frac{1}{V}\int_{A_{{}_{\mathrm{int}}}}cu_{j}n_{j}dA+s$$

where the subscripts *ex* and *in* indicate extracellular fluid and intracellular fluid phases, respectively. Note that the velocity in the intracellular fluid vanished because the integral of the velocity vector in the cell (i.e., closed space) is zero. In contrast, the last term on the right-hand side, which expresses the mass transport with flow through cell membranes (i.e., plasma refilling [24]), cannot be eliminated because of the product of the unit vector. In this analysis, the assumption that the perfusion by plasma refilling via the cell membrane has a uniform velocity vector in the normal direction of the cell membrane was applied. Based on a previous paper [25], the perfusion rate for plasma refilling per unit volume *ν_pl_* (s^−1^) is defined as:

(7) $$\nu_{pl}=\frac{1}{V}\int_{A_{\mathrm{int}}}u_{j}n_{j}dA$$

and the third terms in Equations (5) and (6) can be modeled as:

(8) $$\frac{1}{V}\int_{A_{d_{\mathrm{int}}}}cu_{j}n_{d}{}_{j}dA=\nu_{pl}{\langle c\rangle }^{in}$$

On the other hand, the second term on the right-hand side in Equations (5) and (6), which describes the diffusive mass transport between extracellular and intracellular fluid phases, may be modeled via Newton’s cooling law as:

(9) $$\frac{1}{V}\int_{A_{\mathrm{int}}}D\frac{\partial c}{\partial x_{j}}n_{j}dA=a_{sf}h\left({\langle c\rangle }^{in}-{\langle c\rangle }^{ex}\right)$$

where *a_sf_* is the specific surface area, and *h* is the overall mass transfer coefficient between the extracellular fluid and intracellular fluid phases. Furthermore, the second and third bracketed terms on the right-hand sides of Equations (5) and (6), which describe the effects of tortuosity and mechanical dispersion, respectively, are negligibly small around peripheral tissues. Thus, the macroscopic species mass transfer equations for extracellular fluid and intracellular fluid phases can be expressed as:

(10) $$\frac{\partial \varepsilon_{ex}{\langle c\rangle }^{ex}}{\partial t}+\langle u_{j}\rangle \frac{\partial {\langle c\rangle }^{ex}}{\partial x_{j}}=\frac{\partial }{\partial x_{j}}\left(\varepsilon_{ex}D\frac{\partial {\langle c\rangle }^{ex}}{\partial x_{j}}\right)-a_{sf}h\left({\langle c\rangle }^{ex}-{\langle c\rangle }^{in}\right)+\nu_{pl}{\langle c\rangle }^{in}$$

(11) $$\frac{\partial \varepsilon_{in}{\langle c\rangle }^{in}}{\partial t}=\frac{\partial }{\partial x_{j}}\left(\varepsilon_{in}D\frac{\partial {\langle c\rangle }^{in}}{\partial x_{j}}\right)-a_{sf}h\left({\langle c\rangle }^{in}-{\langle c\rangle }^{ex}\right)-\nu_{pl}{\langle c\rangle }^{in}+s$$

We shall derive the two-compartment models from Equations (10) and (11). Commonly, the measured toxin concentration from a vein during and after dialysis is considered to be the average concentration, rather than the concentration in an individual organ. Therefore, the above macroscopic mass transfer (Equations (10) and (11)) around capillaries is integrated over extracellular and intracellular fluid volumes *V_ex_* and *V_in_,* respectively, with the assumption that the volume of the solute is small enough that it does not affect each volume *V_ex_* and *V_in_*. The lumped parameter models of Equations (12) and (13) can be led as follows:

(12) $$\frac{dV_{ex}\left(t\right)C_{ex}\left(t\right)}{dt}=-Ah\left[C_{ex}\left(t\right)-C_{in}\left(t\right)\right]+\omega_{pl}C_{in}\left(t\right)+B.C.$$

(13) $$\frac{dV_{in}\left(t\right)C_{in}\left(t\right)}{dt}=-Ah\left[C_{in}\left(t\right)-C_{ex}\left(t\right)\right]-\omega_{pl}C_{in}\left(t\right)+S$$

where *C*(*t*) is a function of time *t* and indicates the average concentration over the intrinsic volume of each phase in the body. Therefore, the terms on the left-hand side in Equations (12) and (13) indicate the amount of toxin change per unit of time in the extracellular and intracellular fluid volumes, respectively. On the other hand, the first terms on the right-hand side in Equations (12) and (13) indicate the mass transport that occurs between the extracellular and intracellular fluid phases, where *A* is the total surface area between the extracellular and intracellular fluid phases. Furthermore, *h* is the overall mass transfer coefficient. The second terms on the right-hand side in Equations (12) and (13) indicate the amount of toxin transported with plasma refilling. Here, the perfusion rate of plasma refilling *ω_pl_* (mL/s) was assumed to be a positive value because plasma mainly flows out from the intracellular fluid phase to the extracellular fluid phase during dialysis. Moreover, the third term in Equation (13) indicates the amount of toxin produced in the body, *S* is the value of *s* integrated over the intracellular fluid volume *V_in_*. Therefore, Equations (12) and (13) are a general two-compartment model obtained from mass transport phenomena in the body.

We shall derive analytical solution of Equations (12) and (13). In Equation (12), *B.C.* indicates the mass increase–decrease rate associated with the boundary condition. In the present study, firstly, we shall attempt to analyze the time development toxin concentration in the body during dialysis. The boundary condition during dialysis is given using the clearance *K*:

(14) $$B.C.=-KC_{ex}\left(t\right)$$

This equation indicates the toxin removal rate per unit of time in a dialyzer, which is taken into account in the toxin removal rates by diffusion and ultrafiltration in the dialyzer [26,27].

As shown in Equations (12) and (13), each volume is also a function of time because the intracellular and extracellular volumes change during dialysis. The volumes of the extracellular and intracellular fluids are changed due to ultrafiltration and plasma refilling over the dialysis treatment time as follows:

(15) $$V_{ex}\left(t\right)=V_{ex}\left(0\right)-\left(\omega_{f}-\omega_{pl}\right)t$$

(16) $$V_{in}\left(t\right)=V_{in}\left(0\right)-\omega_{pl}t$$

where *ω_f_* indicates the ultrafiltration flow rate in the dialyzer, for which it is assumed that the ultrafiltration flow rate *ω_f_* is always greater than the perfusion rate of plasma refilling *ω_pl_*. This is because, in dialysis, the plasma feeding occurs due to the decrease in blood volume with ultrafiltration. By substituting Equations (15) and (16) into Equations (12) and (13) and then removing *V* from the time derivatives according to the differential formula rule, the following mass balance equations can be obtained:

(17) $$V_{ex}\left(t\right)\frac{dC_{ex}\left(t\right)}{dt}=-Ah\left[C_{ex}\left(t\right)-C_{in}\left(t\right)\right]+\omega_{pl}C_{in}\left(t\right)+\left(\omega_{f}-\omega_{pl}\right)C_{ex}\left(t\right)-KC_{ex}\left(t\right)$$

(18) $$V_{in}\left(t\right)\frac{dC_{in}\left(t\right)}{dt}=Ah\left[C_{ex}\left(t\right)-C_{in}\left(t\right)\right]+S$$

In the present study, we attempt to derive analytical solutions of the mass balance equations, i.e., Equations (17) and (18). Based on the papers [10,11,12,13,28], the extracellular fluid volume and intracellular fluid volume are assumed to maintain a constant ratio:

(19) $$\varepsilon =\frac{V_{ex}\left(t\right)}{V_{in}\left(t\right)}=const.$$

The term *ω_pl_* can be estimated from Equations (15), (16) and (19) as follows:

(20) $$\omega_{pl}=\frac{\varepsilon V_{in}\left(0\right)-\left[V_{ex}\left(0\right)-\omega_{f}t\right]}{\left(1+\varepsilon \right)t}≅const.$$

From Equation (20), *ω_pl_* is approximately constant under dialysis treatment conditions. Therefore, in the present study, *ω_pl_* was treated as a constant.

During dialysis (0 ≦ *t* ≦ *T*), the extracellular fluid volume and the intracellular fluid volume change over time. Therefore, for obtaining the general solutions of Equations (17) and (18), we essentially use Formulas (19) and (20). In the present study, we introduce a variable transformation. A new parameter, t*, that satisfies the following relationship is introduced:

(21) $$\frac{dt}{dt^{*}}\equiv \frac{df\left(t^{*}\right)}{dt^{*}}\equiv V_{ex}\left(t\right)$$

Thus, from Equations (15) and (21), the following relationship is obtained:

(22) $$dt^{*}=\frac{dt}{V_{ex}\left(0\right)-\left(\omega_{f}-\omega_{pl}\right)t}$$

By integrating this equation under the initial conditions (at *t* = 0: t* = 0), a relational expression between *t* and t* can be obtained as follows:

(23) $$t=\frac{V_{ex}\left(0\right)}{\omega_{f}-\omega_{pl}}\left\{1-\exp\left[-\left(\omega_{f}-\omega_{pl}\right)t^{*}\right]\right\}$$

On the other hand, the concentrations for the extracellular and intracellular fluid phases, which are functions *f* of t*, are defined as:

(24) $$\bar{C_{ex}}\left(t^{*}\right)\equiv C_{ex}\left(f\left(t^{*}\right)\right)=C_{ex}\left(t\right)$$

(25) $$\bar{C_{in}}\left(t^{*}\right)\equiv \frac{1}{\varepsilon }C_{in}\left(f\left(t^{*}\right)\right)=\frac{1}{\varepsilon }C_{in}\left(t\right)$$

Differentiating $\bar{C_{ex}}\left(t^{*}\right)$ and $\bar{C_{in}}\left(t^{*}\right)$ with respect to t*, the following relations can be obtained:

(26) $$\frac{d\bar{C_{ex}}\left(t^{*}\right)}{dt^{*}}=\frac{df\left(t^{*}\right)}{dt^{*}}\frac{dC_{ex}\left(f\left(t^{*}\right)\right)}{df\left(t^{*}\right)}=V_{ex}\left(t\right)\frac{dC_{ex}\left(t\right)}{dt}$$

(27) $$\frac{d\bar{C_{in}}\left(t^{*}\right)}{dt^{*}}=\frac{1}{\varepsilon }\frac{df\left(t^{*}\right)}{dt^{*}}\frac{dC_{in}\left(f\left(t^{*}\right)\right)}{df\left(t^{*}\right)}=V_{in}\left(t\right)\frac{dC_{in}\left(t\right)}{dt}$$

In other words, the differential values of $\bar{C_{ex}}\left(t^{*}\right)$ and $\bar{C_{in}}\left(t^{*}\right)$ correspond to the left-hand sides of Equations (17) and (18), respectively. The right-hand sides of Equations (17) and (18) are similarly rewritten in terms of $\bar{C_{ex}}\left(t^{*}\right)$ and $\bar{C_{in}}\left(t^{*}\right)$, as follows:

(28) $$\frac{d\bar{C_{ex}}\left(t^{*}\right)}{dt^{*}}=-Ah\left[\bar{C_{ex}}\left(t^{*}\right)-\varepsilon \bar{C_{in}}\left(t^{*}\right)\right]+\varepsilon \omega_{pl}\bar{C_{in}}\left(t^{*}\right)+\left(\omega_{f}-\omega_{pl}\right)\bar{C_{ex}}\left(t^{*}\right)-K\bar{C_{ex}}\left(t^{*}\right)$$

(29) $$\frac{d\bar{C_{in}}\left(t^{*}\right)}{dt^{*}}=Ah\left[\bar{C_{ex}}\left(t^{*}\right)-\varepsilon \bar{C_{in}}\left(t^{*}\right)\right]+S$$

Thus, the number of governing equations, i.e., Equations (28) and (29), and the number of unknowns became the same by introducing the defining equations, i.e., Equations (21), (24) and (25), which means that we can solve the analytical solutions. Therefore, in the present study, the analytical solutions are derived from Equations (28) and (29).

From simultaneous Equations (19) and (20), the second-order ordinary differential equation can be obtained as follows:

(30) $$\alpha \frac{d^{2}\bar{C_{ex}}\left(t^{*}\right)}{dt^{*}{}^{2}}+\beta \frac{d\bar{C_{ex}}\left(t^{*}\right)}{dt^{*}}+\gamma \bar{C_{ex}}\left(t^{*}\right)=S$$

where

(31a) $$\alpha =\frac{1}{\varepsilon \left(Ah+\omega_{pl}\right)}$$

(31b) $$\beta =\frac{\left(1+\varepsilon \right)Ah+K-\omega_{f}+\omega_{pl}}{\varepsilon \left(Ah+\omega_{pl}\right)}$$

(31c) $$\gamma =\frac{Ah\left(K-\omega_{f}\right)}{Ah+\omega_{pl}}$$

are constants. Equation (30) can be solved, and the general solution of $\bar{C_{ex}}\left(t^{*}\right)$ is given as follows:

(32) $$\bar{C_{ex}}\left(t^{*}\right)=C_{1}\exp\left(-\lambda_{1}t^{*}\right)+C_{2}\exp\left(-\lambda_{2}t^{*}\right)+\frac{S}{\gamma }$$

where

(33) $$-\lambda_{1or2}=\frac{-\beta^{2}\mp \sqrt{\beta^{2}-4\alpha \gamma }}{2\alpha }$$

and $C_{1}$ and $C_{2}$ are integration constants. In Equation (30), the analytical solution associated with the multiple root is omitted to enhance the understanding of the content for the reader, since it was confirmed that there is probably no multiple root within the range in dialysis treatment by an exhaustive investigation. Furthermore, by substituting Equation (32) into Equation (29), the general solution of $\bar{C_{in}}\left(t^{*}\right)$ is obtained as follows:

(34) $$\bar{C_{in}}\left(t^{*}\right)=\frac{Ah+K-\omega_{f}+\omega_{pl}-\lambda_{1}}{\varepsilon \left(Ah+\omega_{pl}\right)}C_{1}\exp\left(-\lambda_{1}t^{*}\right)+\frac{Ah+K-\omega_{f}+\omega_{pl}-\lambda_{2}}{\varepsilon \left(Ah+\omega_{pl}\right)}C_{2}\exp\left(-\lambda_{2}t^{*}\right)\;+\frac{Ah+K-\omega_{f}+\omega_{pl}}{\varepsilon \left(Ah+\omega_{pl}\right)}\frac{S}{\gamma }$$

By exploiting Equation (23) through Equation (25), the analytical solutions for the toxin concentrations in the extracellular and intracellular fluids during dialysis are derived as follows.

Analytical solution during dialysis:

(35) $$C_{ex}\left(t\right)=C_{1}{\left[1-\frac{\omega_{f}-\omega_{pl}}{V_{ex}\left(0\right)}t\right]}^{\frac{\lambda_{1}}{\omega_{f}-\omega_{pl}}}+C_{2}{\left[1-\frac{\omega_{f}-\omega_{pl}}{V_{ex}\left(0\right)}t\right]}^{\frac{\lambda_{2}}{\omega_{f}-\omega_{pl}}}+\frac{S}{\gamma }$$

(36) $$C_{in}\left(t\right)=\frac{Ah+K-\omega_{f}+\omega_{pl}-\lambda_{1}}{Ah+\omega_{pl}}C_{1}{\left[1-\frac{\omega_{f}-\omega_{pl}}{V_{ex}\left(0\right)}t\right]}^{\frac{\lambda_{1}}{\omega_{f}-\omega_{pl}}}\;+\frac{Ah+K-\omega_{f}+\omega_{pl}-\lambda_{2}}{Ah+\omega_{pl}}C_{2}{\left[1-\frac{\omega_{f}-\omega_{pl}}{V_{ex}\left(0\right)}t\right]}^{\frac{\lambda_{2}}{\omega_{f}-\omega_{pl}}}\;+\frac{Ah+K-\omega_{f}+\omega_{pl}}{Ah+\omega_{pl}}\frac{S}{\gamma }$$

where the integration constants $C_{1}$ and $C_{2}$ can be determined by the initial conditions of the extracellular and intracellular fluid phases. In the present study, we adopted the following initial conditions:

(37) $$t=0:\;C_{ex}\left(0\right)=C_{in}\left(0\right)=C_{S}$$

Thus, $C_{1}$ and $C_{2}$ were estimated as follows:

(38) $$C_{1}=\frac{Ah\left(K-\omega_{f}\right)\left(-K+\omega_{f}+\lambda_{2}\right)C_{s}-\lambda_{2}\left(Ah+\omega_{pl}\right)S}{Ah\left(K-\omega_{f}\right)\left(\lambda_{2}-\lambda_{1}\right)}$$

(39) $$C_{2}=-\frac{Ah\left(K-\omega_{f}\right)\left(-K+\omega_{f}+\lambda_{1}\right)C_{s}-\lambda_{1}\left(Ah+\omega_{pl}\right)S}{Ah\left(K-\omega_{f}\right)\left(\lambda_{2}-\lambda_{1}\right)}$$

Next, we shall derive analytical solution during the process in which intracellular and extracellular toxin concentrations re-equilibrate after dialysis (i.e., rebound process). After dialysis (*t* ≧ *T*), the blood purification in the dialyzer was completed, so *K* = 0 mL/min and $\omega_{f}$ = 0 mL/min. Accordingly, $\omega_{pl}$ is considered to be 0 mL/min. If the patient is not eating or drinking, then the extracellular and intracellular fluid volumes after dialysis are constant. Thus, the general solutions of Equations (17) and (18) can be obtained without introducing variable conversion. From Equations (17) and (18), the second-order ordinary differential equation for evaluating the rebound process can be obtained as follows:

(40) $$\frac{V_{ex}\left(T\right)\cdot V_{in}\left(T\right)}{Ah}\frac{d^{2}C_{ex}\left(t\right)}{dt}+\left[V_{ex}\left(T\right)+V_{in}\left(T\right)\right]\frac{dC_{ex}\left(t\right)}{dt}=S$$

Thus, the general solution of $C_{ex}\left(t\right)$ is given as follows:

(41) $$C_{ex}\left(t\right)=C_{3}\exp\left(-\lambda_{3}t\right)+C_{4}+\frac{S}{V_{ex}\left(T\right)+V_{in}\left(T\right)}t$$

where

(42) $$-\lambda_{3}=-\frac{Ah\left[V_{ex}\left(T\right)+V_{in}\left(T\right)\right]}{V_{ex}\left(T\right)\cdot V_{in}\left(T\right)}$$

where $C_{3}$ and $C_{4}$ are integration constants, and by substituting Equation (41) into Equation (18), the general solution of $\bar{C_{in}}\left(t^{*}\right)$ is given as follows:

(43) $$C_{in}\left(t\right)=\left[1-\frac{V_{ex}\left(T\right)}{Ah}\lambda_{3}\right]C_{3}\exp\left(-\lambda_{3}t\right)+C_{4}+\frac{S}{V_{ex}\left(T\right)+V_{in}\left(T\right)}\left[t+\frac{V_{ex}\left(T\right)}{Ah}\right]$$

where integration constants $C_{3}$ and $C_{4}$ can be estimated based on the initial conditions for the extracellular and intracellular fluid phases. In the present study, the toxin concentrations in the extracellular and intracellular fluids at the end of dialysis $C_{ex}\left(T\right)$ and $C_{in}\left(T\right)$ were adopted as the initial conditions, which were calculated from the analytical solutions, i.e., Equations (35) and (36):

(44) $$t=T:C_{ex}\left(T\right)=C_{ex}{\left(T\right)}_{\begin{array}{cc} during & dialysis \end{array}},\;C_{in}\left(T\right)=C_{in}{\left(T\right)}_{\begin{array}{cc} during & dialysis \end{array}}$$

As a result of this initial condition, $C_{3}$ and $C_{4}$ were estimated as follows:

(45) $$C_{3}=-\frac{1}{\lambda_{3}\exp\left(-\lambda_{3}T\right)}\left[\frac{\left(C_{in}\left(T\right)-C_{ex}\left(T\right)\right)Ah}{V_{ex}\left(T\right)}-\frac{S}{V_{ex}\left(T\right)+V_{in}\left(T\right)}\right]$$

(46) $$C_{4}=C_{ex}\left(T\right)+\frac{\left(C_{in}\left(T\right)-C_{ex}\left(T\right)\right)Ah}{V_{ex}\left(T\right)\cdot \lambda_{3}}-\frac{S}{V_{ex}\left(T\right)+V_{in}\left(T\right)}\left(\frac{1}{\lambda_{3}}+T\right)$$

Combining the analytical solutions during dialysis (i.e., Equations (35) and (36) with Equations (38) and (39)) and the analytical solutions after dialysis (i.e., Equations (41) and (43) with Equations (45) and (46)), it is possible to predict the time development of the toxin concentration in the body during and after dialysis.

## 3. Results and Discussion

### 3.1. Validity of the Proposed Analytical Solutions

Comparison with numerical solutions is performed because the behavior indicated by the proposed analytical solution is unknown, and it is possible to determine whether the solution is valid by comparing the proposed analytical solution with the numerical solution. The numerical solution for the urea concentrations in the extracellular fluid and intracellular fluid phases was obtained by the finite difference calculation based on the mass balance equations, i.e., Equations (17) and (18). In the calculations during dialysis (0 ≦ *t* ≦ *T*), the urea concentration after time step Δ*t* from *t* was predicted by the following difference formulas:

(47) $$C_{ex}\left(t+\Delta t\right)=C_{ex}\left(t\right)-\frac{\Delta t}{V_{ex}\left(t\right)}\left\{KC_{ex}\left(t\right)+Ah\left[C_{ex}\left(t\right)-C_{in}\left(t\right)\right]-\omega_{pl}C_{in}\left(t\right)-\left(\omega_{f}-\omega_{pl}\right)t\right\}$$

(48) $$C_{in}\left(t+\Delta t\right)=C_{in}\left(t\right)+\frac{\Delta t}{V_{in}\left(T\right)}\left\{Ah\left[C_{ex}\left(t\right)-C_{in}\left(t\right)\right]+S\right\}$$

The calculation conditions, which were adopted as the common dialysis conditions in Japan according to a previous study [29], are shown in Table 1. Furthermore, the urine concentrations at the start of dialysis (*t* = 0 min) were set to $C_{s}=C_{ex}\left(0\right)=C_{in}\left(0\right)=80\;\mathrm{mg}/\mathrm{dL}$. The flow rate of ultrafiltration in the dialyzer *ω_f_* was set to 20 mL/min. The perfusion rate of plasma refilling *ω_pl_* is a function of ultrafiltration in the dialyzer *ω_f_* (see Equation (20)), which was estimated to be 12 mL/min. The time step was set to be sufficiently small in order to minimize the numerical error of the numerical simulation.

On the other hand, in the post-dialysis calculations (*t* ≧ *T*), the following difference formulas were adopted:

(49) $$C_{ex}\left(t+\Delta t\right)=C_{ex}\left(t\right)-\frac{Ah\cdot \Delta t}{V_{ex}\left(T\right)}\left[C_{ex}\left(t\right)-C_{in}\left(t\right)\right]$$

(50) $$C_{in}\left(t+\Delta t\right)=C_{in}\left(t\right)+\frac{\Delta t}{V_{in}\left(T\right)}\left\{Ah\left[C_{ex}\left(t\right)-C_{in}\left(t\right)\right]+S\right\}$$

In this calculation after the dialysis, the parameters in Table 1 were also adopted, and the clearance *K* and the perfusion rates of plasma refilling *ω_pl_* and ultrafiltration *ω_f_* in the dialyzer are not needed for this calculation (i.e., *K* = 0 mL/min, $\omega_{f}$ = 0 mL/min, and $\omega_{pl}$ = 0 mL/min). The initial conditions for the analytical and numerical calculations are the urea concentrations at the end of dialysis (*t* = 240 min), which were obtained by analytical and numerical calculations, respectively, during the dialysis. The urea concentrations in the extracellular and intracellular fluid phases for 160 min after dialysis were calculated.

**Figure 3:**
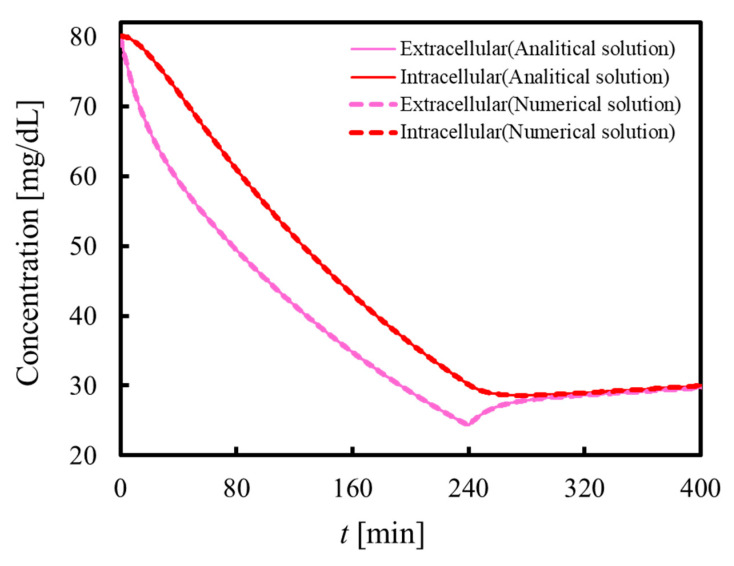
Comparison between analytical and numerical solutions. The time developments of the urea concentration obtained by the analytical solution and the numerical simulation agree well with each other, which proves the validity of the analytical solution.

Figure 3 shows the urea concentrations in the extracellular and intracellular fluid phases during dialysis (0 ≦ *t* ≦ 240 min) and after dialysis (240 < *t* ≦ 400 min), which were estimated from the proposed analytical solutions and numerical simulation, as explained above. As can be seen, the urea concentrations in the extracellular and intracellular fluid phases decrease during dialysis, and the decrease rate in the extracellular fluid phase is faster than that in the intracellular fluid phase. This is because the blood purification in a dialyzer is performed at the blood phase, which is a component of the extracellular fluid. This decrease in the urea concentration in the extracellular fluid causes the difference in the urea concentration between the extracellular and intracellular fluid phases, which induces mass transfer between the extracellular and intracellular fluid phases. Furthermore, plasma refilling occurs from the cells into the extracellular fluid, and urea is removed from intracellular fluid phases with plasma refilling.

In contrast, after dialysis in Figure 3, the urea concentration in the extracellular fluid increases and approaches that of the intracellular fluid. This is because mass transfer between the extracellular and intracellular fluid phases continues after dialysis. The urea concentration in the intracellular fluid phase is higher than that in the extracellular fluid at the end of dialysis. Therefore, urea moves from the intracellular fluid phase to the extracellular fluid phase after dialysis. This phenomenon, in which the blood toxin concentration increases after dialysis, is referred to as rebound, which indicates that the proposed analysis can also predict the rebound phenomenon. Incidentally, the urea concentrations in the extracellular and intracellular fluid phases slightly increase after dialysis because urea is produced in the cells. As shown in Figure 3, the time developments of the urea concentration obtained by the analytical solution and the numerical simulation agree well with each other, which proves the validity of the analytical solution.

### 3.2. Comparison of the Analytical Solution and Clinical Data

In order to prove the usefulness of the proposed analytical solution, the solution must be able to reproduce clinical data. Therefore, using the proposed analytical solutions, we attempted to reproduce the clinical data for blood urea concentration during dialysis (240 min) and after dialysis (60 min) for three chronic dialysis patients, as investigated by Ono et al. [11]. Furthermore, the proposed analytical solution was compared with Gotch formula [1] and Shinzato formula [4], which are the single-compartment model.

In reproducing the clinical data, the clearance of the dialyzer *K*, the volumetric mass transfer coefficient at the cell membrane *Ah*, and the urea production rate *S* were varied to fit the clinical data. Table 2 lists the other constant parameters. The initial concentrations were used for the blood urea concentration at *t* = 0 min based on the blood sampling data. Furthermore, since in the previous study [29], the weight and body water content for each patient were not presented, the average values for Japanese dialysis patients were applied as follows: initial weight 60 kg, body fluid content ratio 60%, and ratio of the extracellular fluid to intracellular fluid phases 4:6. The amount of ultrafiltration in the dialyzer was set such that 4800 mL of water was removed by dialysis for 240 min. Although some of the parameters used in the analysis may differ from the personal data, the adopted values are very commonly used clinically [9,29,30], and verifying the proposed analysis solutions is not problematic. In the reproduction of clinical data by the Shinzato and Gotch formulas, the clearance of the dialyzer in the Shinzato formula $K_{Shinzato}$ and the clearance of the dialyzer in the Gotch formula $K_{Gotch}$ was set so as to match the measured blood urea concentration at the end of dialysis. The toxin concentration of the Shinzato formula is given as follows [4]:

During dialysis (0≤t≤T)

(51) $$C_{Shinzato}\left(t\right)=C_{S}\exp\left(-\frac{K_{Shinzato}t}{V_{entire}}\right)+\frac{S}{K_{Shinzato}}\left[1-\exp\left(-\frac{K_{Shinzato}t}{V_{entire}}\right)\right]$$

After dialysis (t>T)

(52) $$C_{Shinzato}\left(t\right)=C_{Shinzato}\left(T\right)+\frac{S}{V_{entire}}\left(t-T\right)$$

where, $V_{entire}$ is the urea distribution volume, which satisfies a relation $V_{entire}$ = *V_in_*(0) + *V_ex_*(0). $C_{Shinzato}\left(t\right)$ is the urea concentration during and after dialysis, and $C_{Shinzato}\left(T\right)$ is the urea concentration at the end of dialysis. In this study, the urea production rate was treated as a constant value.

On the other hand, the toxin concentration of the *Gotch* formula is given as follows [1]:

(53) $$C_{Gotch}\left(t\right)≅C_{S}\exp\left(-\frac{K_{Gotch}t}{V_{entire}}\right)$$

where, $C_{Gotch}\left(t\right)$ is the urea concentration during dialysis.

**Figure 4:**
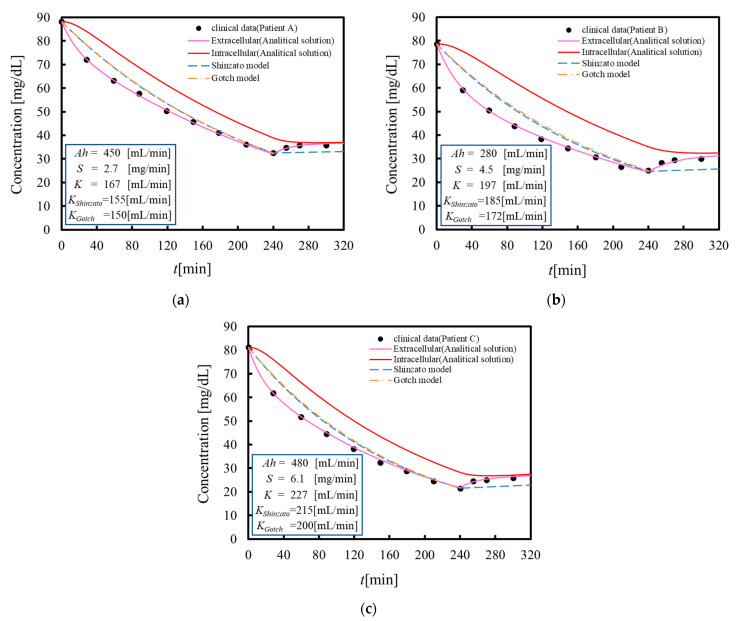
Blood urea concentration reproduced by the proposed analytical solutions, Gotch formula and Shinzato formula. (**a**–**c**) show the case of clinical data of chronic dialysis patients A, B and C, respectively. The proposed analytical solution accurately reproduces the behavior of measured blood urea levels during and after dialysis. This demonstrates the usefulness of the proposed analytical solution.

Figure 4a–c show the blood urea concentration during dialysis (240 min) and after dialysis (60 min) for three chronic dialysis patients reproduced by the proposed analytical solutions, where clinical data are shown for comparison. As shown in Figure 4, the urea concentrations in the extracellular and intracellular fluids decrease at different rates during dialysis and then approach each concentration equilibrium again after dialysis. The proposed analytical solutions accurately reproduce the behavior of measured blood urea concentrations during and after dialysis, including the rebound phenomenon. This indicates the validity of the analytical solution. On the other hand, Figure 4 also shows a comparison of the proposed analytical solution with the Gotch formula during dialysis and with the Shinzato formula during and after dialysis. In the Shinzato and Gotch formulas, there is an error as compared with the measured data in blood urea concentration during and after dialysis. From the viewpoint of accuracy, the proposed analytical solutions may be able to more accurately reproduce the transition of blood urea concentrations, including the rebound phenomenon. Thus, if personal data of the patient, such as the volumetric mass transfer coefficient, are measured by the proposed analytical solutions in advance, the analytical solutions proposed as algebraic expressions allow a doctor to instantly determine the blood toxin concentrations of a patient during and after dialysis. The present model can be easily implemented into a dialysis device, which means that dialysis conditions can be set automatically to be the blood toxin concentration after dialysis desired by the doctor.

## 4. Conclusions

In the present study, a new two-compartment model was derived by adapting volume-averaging theory to the mass transfer around peripheral tissues. Subsequently, the analytical solutions for blood toxin concentration during and after dialysis were proposed by adopting variable transformation. As a set of analytical results, it was found that the urea concentrations in the extracellular and intracellular fluid phases decrease during dialysis, and the decrease rate in the extracellular fluid phase is faster than that in the intracellular fluid phase. Furthermore, after dialysis, the urea concentration in the extracellular fluid increased and approached that of the intracellular fluid, which indicates that the proposed analysis can also predict the rebound phenomenon. Subsequently, comparing the proposed analytical solutions with clinical data for three chronic dialysis patients, it was found that the proposed analytical solutions accurately reproduce the blood urea concentrations during and after dialysis. Furthermore, the proposed analytical solutions may be able to more accurately reproduce the transition of blood urea concentrations as compared with the Gotch and Shinzato formulas.

## Figures and Tables

**Table 1:** Calculation conditions for validation.

***K* [mL/min]**	***T* [min]**	***ε* [-]**	***V_ex_* (0) [mL]**	***V_in_* (0) [mL]**	***C_s_* [mg/dL]**
200	240	0.667	14,400	21,600	80
***ω_f_* [mL/Min]**		***ω_pl_* [mL/Min]**		***Ah* [mL/min]**	***S* [mg/min]**
20		12		500	4.0

**Table 2:** Calculation conditions for comparison with clinical data.

*T* [min]	*ε* [-]	$V_{ex}(0)\;[\mathrm{mL}]$	$V_{in}(0)\;[\mathrm{mL}]$	$\omega_{f}\;[\mathrm{mL}/\min]$	$\omega_{pl}\;[\mathrm{mL}/\min]$
240	0.667	14,400	21,600	20	12
